# Systemic and Intra-Nodal Activation of NK Cells After Rituximab Monotherapy for Follicular Lymphoma

**DOI:** 10.3389/fimmu.2019.02085

**Published:** 2019-09-12

**Authors:** Monika Enqvist, Benedikt Jacobs, Henna R. Junlén, Marie Schaffer, Christopher M. Melén, Danielle Friberg, Björn Engelbrekt Wahlin, Karl-Johan Malmberg

**Affiliations:** ^1^Department of Medicine, Center for Infectious Medicine, Huddinge, Karolinska Institutet, Stockholm, Sweden; ^2^K.G. Jebsen Center for Cancer Immunotherapy, Institute of Clinical Medicine, University of Oslo, Oslo, Norway; ^3^Department of Cancer Immunology, Institute for Cancer Research, Oslo University Hospital, Oslo, Norway; ^4^Department of Hematology and Oncology, University Hospital Erlangen, Erlangen, Germany; ^5^Division of Hematology, Department of Medicine, Huddinge, Karolinska Institutet, Stockholm, Sweden; ^6^Center for Hematology, Karolinska University Hospital, Stockholm, Sweden; ^7^Department of Oto-Rhino-Laryngology, Karolinska University Hospital and CLINTEC, Karolinska Institutet, Stockholm, Sweden

**Keywords:** NK cell, follicular lymphoma (FL), rituximab, killer cell immunoglobin-like receptor, ki67

## Abstract

Monotherapy with the anti-CD20 monoclonal antibody rituximab can induce complete responses (CR) in patients with follicular lymphoma (FL). Resting FcRγIII^+^ (CD16^+^) natural killer (NK) cells respond strongly to rituximab-coated target cells *in vitro*. Yet, the contribution of NK cells in the therapeutic effect *in vivo* remains unknown. Here, we followed the NK cell repertoire dynamics in the lymph node and systemically during rituximab monotherapy in patients with FL. At baseline, NK cells in the tumor lymph node had a naïve phenotype albeit they were more differentiated than NK cells derived from control tonsils as determined by the frequency of CD56^dim^ NK cells and the expression of killer cell immunoglobulin-like receptors (KIR), CD57 and CD16. Rituximab therapy induced a rapid drop in NK cell numbers coinciding with a relative increase in the frequency of Ki67^+^ NK cells both in the lymph node and peripheral blood. The Ki67^+^ NK cells had slightly increased expression of CD16, CD57 and higher levels of granzyme A and perforin. The *in vivo* activation of NK cells was paralleled by a temporary loss of *in vitro* functionality, primarily manifested as decreased IFNγ production in response to rituximab-coated targets. However, patients with pre-existing NKG2C^+^ adaptive NK cell subsets showed less Ki67 upregulation and were refractory to the loss of functionality. These data reveal variable imprints of rituximab monotherapy on the NK cell repertoire, which may depend on pre-existing repertoire diversity.

## Introduction

Follicular Lymphoma (FL) is the second most common non-Hodgkin's lymphoma and accounts for 20% of all lymphomas. Most patients respond well to series of treatment strategies, but the relapse rate is high and the disease is in the majority of cases incurable (1). Since the initial demonstration that monotherapy with rituximab, an antibody recognizing the CD20 antigen expressed on mature B cells, could improve the survival in FL (2–4), immunotherapy has become a corner stone in the therapy of most B cell lymphomas. One of the major mechanisms of action for rituximab is antibody-dependent cellular cytotoxicity (ADCC), which is mediated by natural killer (NK) cells and/or macrophages (5, 6). NK cells perform ADCC through engagement of an FcγRIIIA/CD16 receptor to the Fc-part of antibodies bound to the target cell. This process is known to trigger strong NK cell activation through different pathways including release of cytotoxic granules and pro-inflammatory cytokines as IFNγ (7).

The involvement of NK cells in the clinical effects of rituximab is indirectly supported by the findings that NK cells are rapidly cleared from the circulation after therapy with rituximab (8), and the correlation with polymorphisms in the FcγRIIIA gene (5, 9, 10). In many clinical studies it is challenging to determine the isolated immunological response to rituximab because of different treatment combinations. In one study the outcome in non-Hodgkin's lymphoma patients correlated with expansion of NK cells and increased ADCC after the patients received IL-2 in addition to rituximab therapy (11). Functional *in vitro* studies have shown that rituximab activates a broad range of NK cell subsets, independently of their expression of self-HLA binding inhibitory KIRs, thus overriding the need for education (12). On the other hand, it has been reported that tumor cells can increase HLA class I expression in response to IFNγ stimulation and thereby escape NK cell killing. However, the *in vivo* dynamics of the NK cell repertoire, both systemically and in the lymph node, during monotherapy with rituximab is largely unexplored. In this study, we examined the immune repertoire in sequential biopsies of the affected lymph node and in peripheral blood in FL patients receiving monotherapy with rituximab. Our results point to a diversified immunological response where some patients display a pronounced up-regulation of Ki67 associated with a temporary drop in NK cell function. The kinetics of the response was linked to the presence of adaptive NK cell subsets in the patient and may hold clues to clinical responsiveness to antibody therapy.

## Results

### NK Cell Frequency and Phenotype in Lymph Node and Peripheral Blood

**Figure 1:**
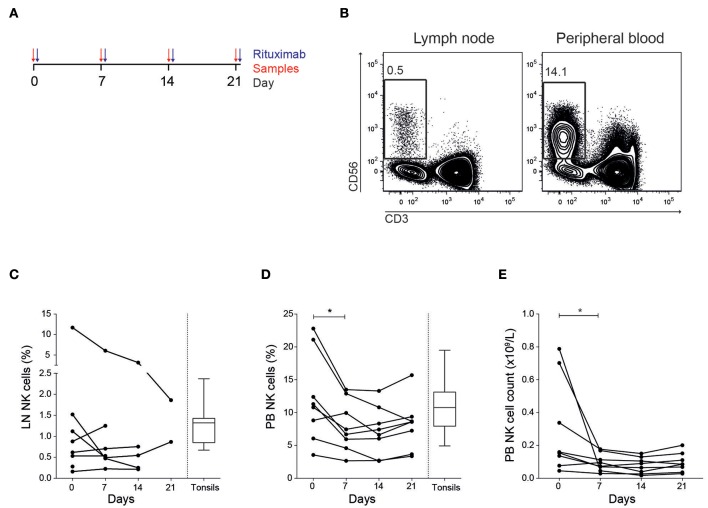
Decreased NK cell frequency and cell count in peripheral blood after treatment. **(A)** Outline of treatment and sample collection in the patients. **(B)** Representative flow cytometry staining of CD56^+^ CD3^−^ NK cells in tumor lymph node and peripheral blood. Frequency CD56^+^ CD3^−^ NK cells of total tumor negative and CD45^+^ cells in **(C)** lymph node (LN) and **(D)** peripheral blood (PB). **(E)** NK cell counts in PB. Patients *N* = 8, healthy controls *N* = 10. Differences were assessed using the Wilcoxon signed rank test for comparisons of matched samples within patients or Mann-Whitney *t*-test for comparisons between healthy controls and patients, *p < 0.05.

Eight patients diagnosed with follicular lymphoma were included in the study (Table 1). All patients were previously untreated and received in total four doses of rituximab (Figure 1A). We first established multi-color flow cytometry panels to monitor the immune subset composition in fine needle biopsies from tumor lymph nodes (LN) and peripheral blood (PB) before each treatment cycle at a weekly interval. The biopsy sample collection continued until the tumor lymph nodes were too small to access. The NK cell frequency in LN samples were consistently low compared to frequencies seen in PB (Figures 1B,C), with patients showing both increasing and decreasing trends over time. However, the relative LN-NK frequency of total CD45^+^ and CD19^−^ CD20^−^ cells were similar to what we found in tonsil samples from healthy donors. In agreement with earlier studies (13), we found a decrease of NK cells in peripheral blood 7 days after rituximab treatment started manifested as lower frequencies and lower absolute counts (Figures 1D,E).

**Table 1:** Patient characteristics.

**ID**	**Age**	**Diagnosis**	**Stage**	**FLIPI**	**Treatment indication**	Response^*^	Response^**^	**Months in remission^+^**	**Subsequent treatments^**#**^**
RITFL01	60–65	FL low grade	IV	Int	High tumor burden, B symptoms	PR	CR	5	R-Idelalisib, R-Bendamustine^#^
RITFL02	30–35	FL 3A	III	Low	Pain in enlarged lymph nodes	CR	CR	68^+^	
RITFL03	35–40	FL 2	IV	High	High tumor burden, B symptoms	PD			R-CHOP, R-Bendamustine, BEAM^+^ ASCT^#^
RITFL04	65–70	FL 2	IV	High	Progressive disease	PR	CR	61^+^	
RITFL05	40–45	FL 2	III	Int	Progressive disease, high tumor burden	PR	PR	17	R^#^
RITFL06	70–75	FL 2	III	High	Progressive/bulky disease	PR	PR	16	R-Bendamustine^#^
RITFL07	70–75	FL low grade	IV	High	Progressive disease	PR	CR	57^+^	
RITFL08	80–85	FL low grade	III	Low	Progressive disease	PR	CR	10	R-Bendamustine^#^

FLIPI, Follicular Lymphoma International Prognostic Index; PR, partial remission; CR, complete remission; R, rituximab; CHOP, cyclophosphamide, doxorubicine, vincristine and prednisolon; BEAM, carmustine, etoposide, cytarabine and melphalan; ASCT, autologous stem cell transplantation.

* After 10 weeks (1 × 4 rituximab);

** After 22 weeks (2 × 4 rituximab);

+ Ongoing remission;

# *Alive at latest follow up*.

**Figure 2:**
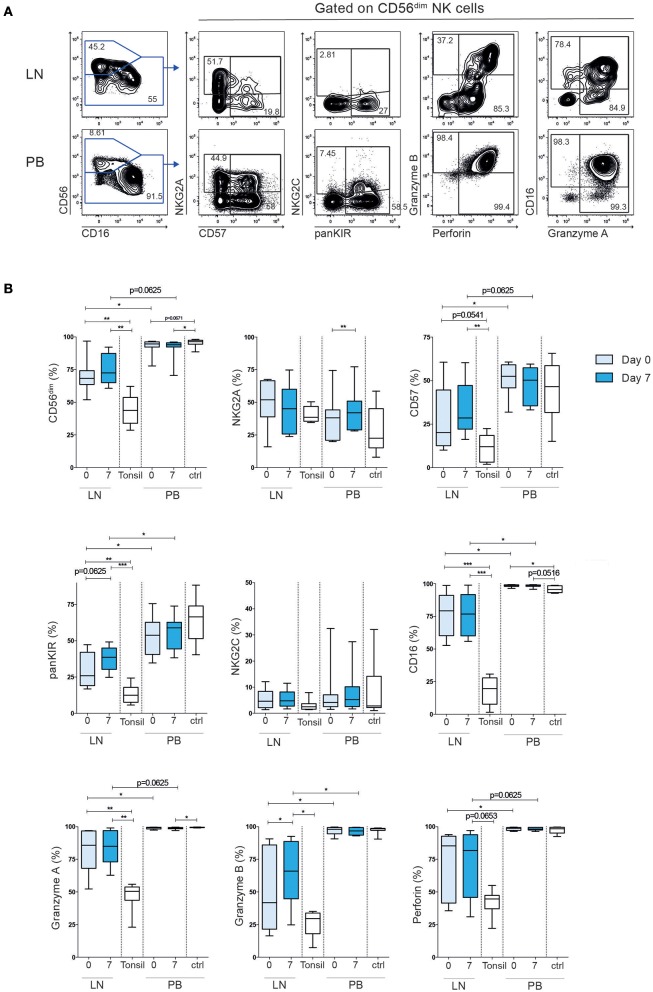
Tumor lymph node NK cells have an intermediate differentiated phenotype compared to tonsil NK cells and peripheral blood NK cells. **(A)** Example of flow cytometry staining of NK cells in tumor lymph node and peripheral blood. **(B)** Frequency CD56^dim^ NK cells of total CD56^+^ cells, and frequency NKG2A^+^, CD57^+^, KIR^+^, NKG2C^+^, CD16^+^, Granzyme A^+^, Granzyme B^+^, Perforin^+^ cells of total CD56^dim^ cells in patient tumor lymph node samples and peripheral blood, before (light blue) and 7 days (dark blue) after treatment. Tonsil and peripheral blood NK cells from healthy controls are shown in white bars. Patients *n* = 8, healthy controls *n* = 8–10. Differences were assessed using the Wilcoxon signed rank test for comparisons of matched samples within patients or Mann-Whitney *t*-test for comparisons between healthy controls and patients, *p < 0.05, **p < 0.01, and ***p < 0.001.

Next, we determined the expression of activating and inhibitory receptors, including killer cell immunoglobulin-like receptors (KIR), NKG2A and NKG2C, effector molecules and maturation markers on intra-nodal and peripheral blood NK cells (Figure 2). In line with previous findings (14, 15), we observed a dominance of CD56^brigh^ NK cells in tonsils from healthy donors (average 56%, range 37–71%). Tonsils are widely used as a control in FL (16, 17), albeit they represent a more inflamed tissue compared to normal lymph nodes from healthy individuals. Tonsils contain more differentiated T cells and are more similar to FL tumors in terms of immune cell composition and differentiation states (18). Indeed, compared to normal tonsils, LN-NK cells in FL patients showed an intermediate phenotype, with an average of 71% CD56^dim^ cells. This intermediate state was also reflected in the relative expression of CD57, KIRs and CD16 on CD56^dim^ NK cells when compared to the same subset in tonsil-derived NK cells and PB-NK cells (Figure 2B). Although we cannot formally exclude that ILCs contributed to the relative composition in CD56+CD3- cells in LN and tonsils, ILCS typically lack CD16, KIR and NKG2A. Furthermore, LN-NK cells expressed lower levels of the effector molecules Granzyme A/B and Perforin than PB-NK cells. The maturation and cytotoxic phenotypes of both LN-NK and PB-NK cells were highly stable over time with the exception of increased Granzyme B in LN-NK after treatment and a small increase of NKG2A^+^ cells in PB-NK cells 7 days after the first rituximab administration. We can not formally exclude that dynamics in ILCs contributed to the

### Rituximab Therapy Is Associated With a Rapid Induction of Ki67 Expression

**Figure 3:**
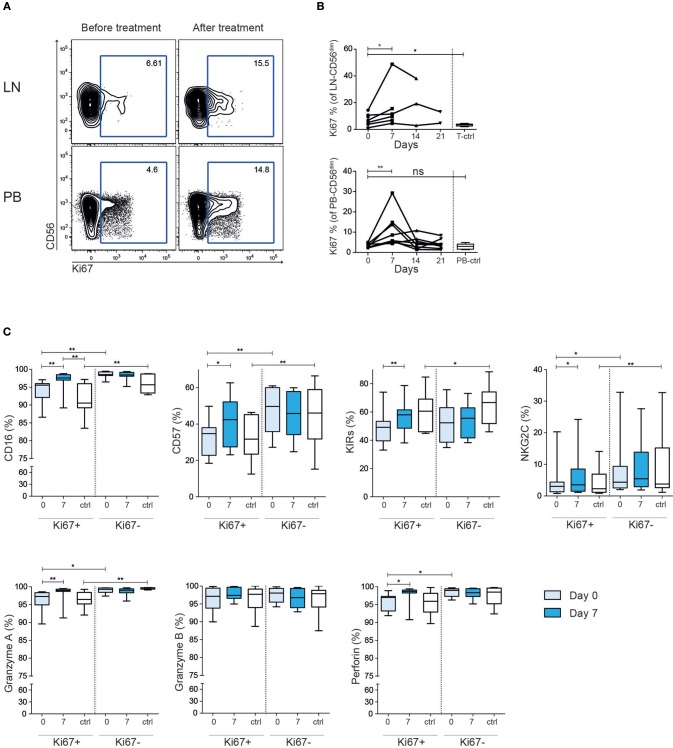
Increased frequency of Ki67^+^ CD56^dim^ NK cells after rituximab treatment. **(A)** Representative example of flow cytometry staining and **(B)** summary showing frequency Ki67^+^ CD56^dim^ NK cells before (light blue) and after (dark blue) treatment in tumor lymph node and peripheral blood. **(C)** Expression of NKG2A, CD57, KIRs, NKG2C, CD16, granzyme A/B and perforin on Ki67 positive and negative CD56^dim^ NK cells from peripheral blood Healthy controls are shown in white. Patients *N* = 8, healthy controls *N* = 10. Differences were assessed using the Wilcoxon signed rank test for comparisons of matched samples within patients or Mann-Whitney *t*-test for comparisons between healthy controls and patients, *p < 0.05 and **p < 0.01.

To further dissect the NK cell response to rituximab treatment in the patients we stained for the intracellular marker Ki67, which is found in recently divided or cycling cells. Before treatment the Ki67 level in the patients' LN-NK cells was higher than tonsil control NK cells, while PB-NK cells had comparable level to what was seen in healthy controls (Figures 3A,B). Seven days after treatment start, we found a consistent increase of Ki67 expression in both LN-NK and PB-NK. After 14 days the Ki67 expression had, in most instances, declined to baseline levels and remained low during the subsequent administration of rituximab. This indicates the observed induction of Ki67 on NK cells in the patients was a direct consequence of the rituximab treatment but that this response was limited to the first administration. The Ki67^+^ NK cells at day 7 expressed relatively higher levels of CD16, CD57, KIRs, NKG2C, Perforin and Granzyme A than Ki67^+^ NK cells at baseline, suggesting that NK cell activation was accompanied with cellular differentiation (Figure 3C).

### Temporary Loss of NK Cell Function After Rituximab Treatment

**Figure 4:**
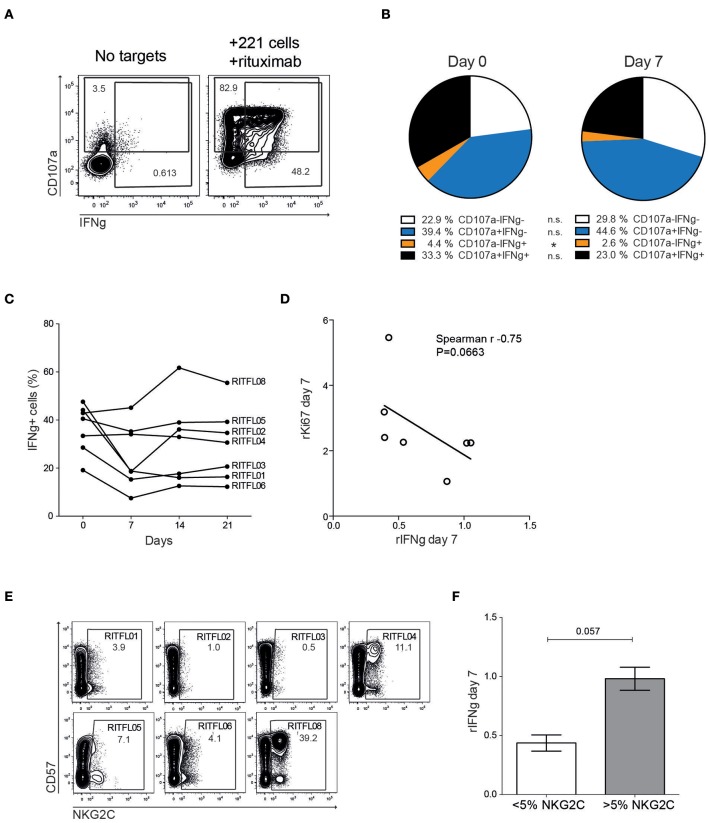
Altered function of peripheral blood CD56^dim^ NK cells after treatment. **(A)** Flow cytometry staining of CD107a and IFNγ on peripheral blood NK cells from patients, with or without tumor cell and rituximab stimulation. **(B)** Relative frequency of CD107a and IFNγ positive CD56^dim^ NK cells after stimulation with 721.221 cells coated with rituximab, in patients (*n* = 7) before (Day 0) and after (Day 7) treatment. **(C)** CD56^dim^ NK cell IFNγ response to 721.221 cells coated with rituximab in the individual patients before and during the first 3 weeks after therapy (*N* = 7). **(D)** Correlation of relative IFNγ response to relative Ki67 expression (day 7 compared to day 0). **(E)** Flow cytometry staining showing NKG2C and CD57 expression in the individual patients at Day 0. (**F)** Relative IFNγ-production at day 7 in patients with less or more than 5% NKG2C^+^ CD56^dim^ NK cells.

To assess whether the repertoire dynamics was associated with changes in NK cell function, we monitored degranulation (CD107a) and cytokine production (IFNγ) in peripheral blood NK cells after stimulation with 721.221 cells coated with rituximab (Figure 4A). We found a reduction in multi-functional responses, primarily due to a significantly decreased IFNγ-production at day 7 after systemic rituximab treatment in some but not all patients (Figures 4B,C). NK cell function gradually returned to normal over the course of 2–3 weeks. Notably, three of the patients showed an opposite trend with a more stable or even increased NK cell function at day 7 and beyond. The relative loss of IFNγ-production appeared to be most prominent in patients that had a relatively higher induction of Ki67^+^ NK cells at day 7 (Figure 4D).

Approximately 30–40% of CMV seropositive donors harbor expansion of adaptive NK cells expressing high levels of NKG2C and CD57 (19, 20). This specific NK cell subset displays epigenetic imprinting of the IFN promoter and responds strongly to antibody-coated targets (21–23). Although the number of patients in the study is small, stratifying the patients into two groups based on presence of NKG2C expansions above 5% revealed a trend toward a more stable IFNγ-production over time in patients with adaptive NKG2C^+^ cells (Figures 4E,F).

### Rituximab Pretreatment Reduces Antibody-Dependent NK Cell Functions *in vitro*

**Figure 5:**
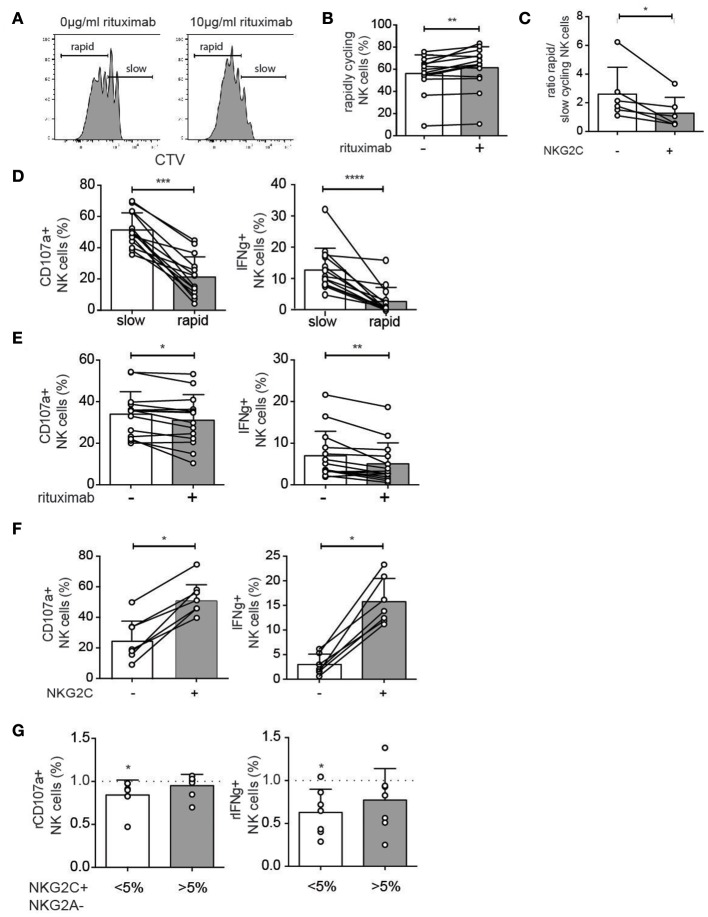
*In vitro* model for altered NK cell functions upon rituximab pre-treatment. **(A,B)** NK cells from CMV-seropositive healthy donors were cultured for 6 days with low-dose IL-15 (1 ng/ml) in the presence or absence of rituximab (10 μg/ml) for the first 48 h. The percentage of rapidly cycling NK cells (at least 2 divisions) was analyzed after 6 days of IL-15 culture with (+) or without (–) rituximab pretreatment. **(C)** The ratio of rapid versus slowly cycling NK cells as defined in panel A, stratified based on expression of NKG2C. **(D)** Degranulation (CD107a) and cytokine production (IFNγ) after stimulation with rituximab-coated 721.221 cells in rapidly and slowly cycling NK cells. **(E)** Degranulation (CD107a) and cytokine production (IFNγ) after stimulation with rituximab-coated 721.221 cells in bulk NK cells with (+) or without (–) rituximab pretreatment *in vitro*. **(F)** Degranulation (CD107a) and cytokine production (IFNγ) after stimulation with rituximab-coated 721.221 cells in NKG2C+ and NKG2C- NK cells. **(G)** Healthy donors were divided into those with (>5%) or without (<5%) a significant expansion of NKG2C^+^ NK cells. The relative degranulation (rCD107a) and relative IFNγ production (rIFNγ) in response to ADCC stimulation for cells with or without pretreatment with rituximab. Statistical significance between paired samples was calculated using a Wilcoxon test and indicated on bars, whereas a Wilcoxon singed-rank test was used to calculate the statistical significance against a fixed value. Non-paired samples were analyzed by a Mann-Whitney *t*-test (*p < 0.05, **p < 0.01, ***p < 0.001, and ****p < 0.0001).

To further study the effect of rituximab on NK cell proliferation and ADCC function PBMCs from healthy donors were cultured with 1 ng/ml IL-15 to induce proliferation, in the presence or absence of a short term (48 h) stimulation with rituximab (10 μg/ml). Rituximab pretreatment resulted in a small but significant increase in the proportion of rapidly cycling NK cells at day 6 (Figures 5A,B). Under these experimental conditions, adaptive NKG2C^+^ NK cells proliferated less than conventional NK cells (Figure 5C). In agreement with the *ex vivo* data, degranulation and cytokine production in response to antibody-coated target cells were reduced in rapidly proliferating NK cells (Figure 5D) and in cells with prior exposure to rituximab (Figure 5E). In line with their low-grade proliferation, NKG2C^+^ NK cells displayed higher functional responses after rituximab stimulation (Figure 5F). Hence, at the repertoire level, IFNγ production was more stable in donors with pre-existing CMV-driven expansions of NKG2C^+^ NK cells (Figure 5G).

## Discussion

NK cells are believed to contribute to the successful eradication of malignant B cells following rituximab therapy, but the mechanism for this is not well-defined. In this study we performed a detailed analysis of the dynamics including functional and phenotypical characteristics of the NK cell response to rituximab treated FL-patients. NK cells in tumor-affected lymph nodes had an immature phenotype, albeit more differentiated than tonsil NK cells used as negative control. Both lymph node and peripheral blood NK cells showed signs of activation/proliferation during the first week after initiation of rituximab treatment. These phenotypic imprints were linked to a temporary loss in functionality. These early dynamic events may hold clues to the role of NK cell-mediated ADCC in the clinical response to therapeutic monoclonal antibodies.

There are surprisingly few studies assessing the NK cell compartment in primary human lymph nodes and tonsils and to our knowledge there is none describing dynamic events following immunomodulatory therapy. Healthy human tonsils and lymph nodes were found to contain 5 and 0.4% NK cells, respectively (15). It was previously reported that 95% of lymph node NK cells are CD56^bright^ (14). We confirm these low numbers of NK cells in lymphoid tissues although NK cell frequencies were similarly low in tumor lymph node biopsies and healthy control tonsils. However, the tumor-affected lymph nodes contained relatively higher numbers of CD56^dim^ NK cells. In addition, several surface receptors associated with a mature cytotoxic phenotype, including CD16, CD57, and KIRs, were expressed at a higher level in tumor-affected lymph nodes compared to tonsil controls. This could reflect an ongoing immunological response to the tumor cell growth.

Besides cytotoxic effector functions, activated NK cells can produce high levels of cytokines, which may in turn lead to further activation of several immune cell types including T cells (8). Previous studies have shown that cytotoxic lymphocytes predominantly reside at the periphery of follicles and in the interfollicular areas close to T cells (24). In this compartment, the interplay with T cells secreting IL-2 leads to NK cell-mediated release of IFNγ (14). IFNγ release can lead to up-regulation of HLA molecules and thereby affect the T cell response, it also stimulates macrophages to increased phagocytosis in addition to lymphocyte recruitment and a prolonged activation (25). An increased CD8^+^ T-cell infiltrate correlates to better prognosis in FL (26) although chronic stimulation of intra-nodal T cells may also lead to T-cell exhaustion in FL patients (27, 28).

Release of pleiotropic cytokines by LN-NK cells in response to rituximab can furthermore influence the local immune response through increased expression of the high-affinity Fcγ receptors CD64 on granulocytes (29) and through M1-polarization of macrophages (30). *In vitro* studies have suggested rituximab to induce cytotoxic T cell response after promoting phagocytosis and dendritic cell cross-presentation (31). Indeed, anti-CD20 therapy led to a long-lasting protection against CD20+ tumor cells in mice by inducing a CD8+ and CD4+ dependent cellular immune response (32). Similarly, vaccination with autologous DC following low dose of intra-nodal rituximab was associated with CR and induction of systemic immunity in patients with FL (33).

We noted a rapid but temporary decline in NK cell numbers in the periphery after rituximab therapy. Unfortunately it was not possible to trace the fate of these cells and determine whether they egressed from peripheral blood to reach other compartments. We did not detect any reciprocal increase in NK cell frequencies in the lymph node. It is possible that we missed the kinetics of these early events by sampling the lymph node weekly. However, Ki67 expression was induced on both PBL-NK and LN-NK 1 week after the first dose of rituximab. The NK cell number in lymph node biopsies was too low to allow further subset analysis of the emerging Ki67^+^ NK cell population. In PBL, Ki67^+^ NK cells had a slightly more differentiated phenotype at day 7 than day 0 and compared to Ki67^+^ NK cells in healthy controls. These data suggest that rituximab triggers a rapid proliferative NK cell response associated with cellular differentiation. Intriguingly, the phenotypic imprints were normalized and had returned to baseline at day 14 in most patients. Also, the NK cell repertoires were stable following the 2nd to 4th treatment cycle. In one of the patients we noted a slower kinetics with a peak in Ki67 expression occurring at day 14. This slow kinetics correlated with a delayed clinical response to the treatment in this particular patient.

In the majority of the patients we observed an attenuated response to *in vitro* stimulation with rituximab-coated target cells 1 week after the first treatment cycle. This drop in functionality coincided in time with the increased expression of Ki67. In fact, we noted a positive correlation between the degree of Ki67 upregulation at day 7 and loss of IFNγ production *in vitro*. Ki67 may reflect a more general NK cell activation but it is also possible that these cells have undergone recent cell division. Onset of cell division and the associated changes in metabolism may reprogram the functionality of the cell leading to decreased cytokine production. Therefore, it is tempting to speculate that the decreased *in vitro* functionality observed at day 7 represent a form of functional exhaustion following the primary *in vivo* response to systemic rituximab therapy. If this is the case, variation in the *in vitro* response may hold clues to the efficacy of the therapy. Supporting this notion, one *in vitro* study reported hyporesponsiveness in NK cells following pre-treatment with rituximab (34). In this short-term model, exposure to rituximab primarily affected cytotoxicity while no effect was seen on the IFNγ production. In contrast, our data showed altered cytokine production while the degranulation toward rituximab-coated target cells was less affected. Furthermore, we found stable or increased levels of the cytotoxic effector molecules granzyme A/B and perforin after treatment, suggesting the ability to perform cellular cytotoxicity remained intact. In a few patients we observed an opposite trend with a stable or even increased functional response at day 7. Intriguingly, these patients had evidence of a weaker systemic imprint of rituximab therapy and harbored significant adaptive NK cell populations determined by the expression of NKG2C and CD57. Adaptive NK cells have increased capacity to produce IFNγ in response to antibody-coated target cells due to epigenetic changes in the IFNγ promoter (21–23, 35). Patients having NKG2C^+^ NK cells could theoretically thus already from the start be better equipped for cytokine production in response to rituximab treatment thereby explaining their functional stability over time. Although these data are based on few patients, this notion was supported by an *in vitro* model for rituximab-induced proliferation where we noted a profound loss of function in proliferating NK cells that was less dramatic in donors harboring adaptive NK cell expansions, linked to the poor proliferative response and maintained function in NKG2C^+^ NK cells.

The primary objective of our study was to explore longitudinal phenotypic and functional imprints of rituximab monotherapy. The study was underpowered to link immune variation to clinical outcomes. Nevertheless, the diversified response observed in this study suggest that probing dynamic systemic and local immune imprints may hold utility as a metrics of the patients' ability to respond to therapy and thereby a predictor treatment efficacy.

## Materials and Methods

### Patients

This study was approved by the Regional Ethical Review Board in Stockholm, Sweden. Fine needle biopsies from tumor lymph nodes and peripheral blood were collected after informed consent from 8 patients diagnosed with follicular lymphoma. Samples were obtained before treatment, and 7, 14, and 21 days after the treatment with rituximab started. Collection of the fine needle biopsies ended earlier if the tumor lymph nodes were too small to access. Age matched healthy control cells were collected from buffy coats and tonsils. The cells from peripheral blood and tonsils were enriched by density gradient (Ficoll-Hypaque; GE Healthcare) and PBMC's, cells from tonsils and fine needle biopsy cells were cryopreserved in 90% FCS and 10% DMSO for later analysis. CMV serology was determined using an ELISA-based assay on plasma obtained during sample preparation. Purified nuclear antigen (AD 169) was used and the cut-off level for seropositivity was an absorbance of >0.2 dilution of 1/100.

### Antibodies and Flow Cytometry

The following conjugated monoclonal antibodies (mAbs) were used: Ki67 (clone: B56), CD14 (MϕP9), CD57 (NK-1), Granzyme B (GB11), CD107a (H4A3), IFNγ (B27), CD19 (HIB19) from BD Biosciences, NKG2A (z199), CD3 (UHCT1), CD56 (N901), KIR2DL2/3/S2 (GL183), KIR2DL1/S1 (EB6), CD19 (J3-119) from Beckman Coulter, CD16 (3G8), CD20 (2H7), CD45 (HI30), Granzyme A (CB9), KIR3DL1 (dx9) from BioLegend, Perforin (dG9), κ-chain (TB28-2), λ-chain (TB28-2), Granzyme K (G3H69) from eBiosciences, KIR2D-biotin (NKVFS1), KIR3DL1/2-biotin (5.133), KIR2DL1 (REA284) from Miltenyi, NKG2C (134591) and 2DL3 (180701) from R&D Systems. For phenotypic analysis of cells, PBMCs and cells from fine needle biopsies were incubated at 4°C in the dark with surface mAbs, washed with PBS and additionally stained with Streptavidin-Qdot655 (Invitrogen) and Live/Dead fixable aqua dead cell stain (Invitrogen). After wash with PBS the cells were fixed and permeabilized with fix/perm kit (eBiosciences), washed and stained with intracellular mAbs. Data were acquired in FACSDiva software on a BD LSRFortessa equipped with a 488-nm laser, a 633-nm laser, a 405-nm laser, and a 562-nm laser. Acquired data were analyzed in FlowJo 9.8 (Tree Star).

### Functional Flow Cytometry Assay

PBMCs were rested overnight at 37°C and 5% CO_2_ in RPMI 1640 supplemented with 10% FCS and 5 mM L-glutamine. Cells were mixed with 721.221 target cells at a 10:1 ratio and incubated 6 h with or without rituximab (1 μg/ml). CD107a antibody, GolgiStop (1:1,500, BD Biosciences) and GolgiPlug (1:1,000, BD Biosciences) were added after 1 h of co-incubation. At the end of the assay additional CD107a antibody was added together with the surface mAbs, following staining procedure as described above.

### *In vitro* Experiments

PBMCs were isolated from buffy coats obtained from healthy blood donors from the Oslo University Hospital Blood bank (donor informed consent included). Cells were labeled with CellTrace™ Violet or CFSE ™ dye for cell proliferation analysis according to the kit's instructions (Molecular Probes). CTV-/CFSE -labeled PBMCs were culture in RMPI 1640 media (Sigma) with antibiotics (penicillin/ streptomycin; Sigma) and 10% human, heat-inactivated AB serum (Trina Bioreaktives) plus 1 ng/ml IL-15 (Miltenyi Biotec) for 6 days at 37°C with or without 10 μg/ ml rituximab (Roche) for the first 48 h. On day 2 and 4 the medium was replaced with fresh medium and IL-15. After 6 days cells were analyzed for the number of dividing NK cells by flow cytometry. In addition, cells were mixed with 721.221 target cells, which have been labeled with or without rituximab, at a 1:1 ratio and incubated for 4 h at 37°C. CD107a antibodies were added directly, whereas GolgiStop (1:1,500, BD Bioscience) and GolgiPlug (1:1,000, BD Bioscience) were added after 1 h of co-incubation. At the end of the culture, cells were stained with surface and intracellular mAbs, following staining procedure as describe above.

### Statistical Analysis

For comparisons Wilcoxon matched test or for unpaired groups Mann-Whitney test was used. *p*-values: ^*^ < 0.05, ^**^ < 0.01, ^***^ < 0.001, ^****^ < 0.0001. Analysis was performed using GraphPad Prism 6 software.

## Data Availability

All data generated and/or analyzed during the current study are available on reasonable request (kalle.malmberg@ki.se).

## Ethics Statement

The studies involving human participants were reviewed and approved by the regional ethics committee in Stockholm, Sweden. The regional ethics committee in Oslo, Norway. The patients/participants provided their written informed consent to participate in this study.

## Author Contributions

ME and BJ conducted experiments and analyzed the data. MS performed HLA and KIR typing. HJ, CM, and DF contributed with clinical samples and biobanking. ME, BW, and K-JM designed research and wrote the manuscript. K-JM is a scientific advisor and consultant at Fate Therapeutics.

### Conflict of Interest Statement

The authors declare that the research was conducted in the absence of any commercial or financial relationships that could be construed as a potential conflict of interest.
